# Supplementary material for: Techno-economic assessment and innovative production of nutrient-rich jam, jelly, and pickle from Sonneratia apetala fruit
Source: PLoS One. 2024 Dec 4;19(12):e0311846. doi: 10.1371/journal.pone.0311846 (PMC11616811; doi:10.1371/journal.pone.0311846)
Supplement: S1 Table — (DOCX) [file pone.0311846.s001.docx]

Table S1: Comparison of different fruits nutrients values and vitamin C

| Fruits | Vitamin C  (mg/100 gm) | Major elements (ppm) | | | | Trace elements (ppm) | |
| --- | --- | --- | --- | --- | --- | --- | --- |
|  |  | Na | K | Mg | Ca | Zn | Cu |
| Lemon | 44.5 | 20 | 1160 | 70 | 220 | 0.5 | 0.31 |
| Lime | 19.5 | 10 | 680 | 40 | 220 | 0.7 | 0.44 |
| Orange | 69.7 | - | 2370 | 130 | 520 | 0.9 | 0.59 |
| Grape fruit | 79.1 | - | 3200 | 180 | 280 | 1.6 | 1.08 |
| *S.apetala* fruit | 100.71 | 9274.2 | 17425.5 | 1440 | 2714.29 | 20.80 | 11.11 |
