# Supplementary material for: Techno-economic assessment and innovative production of nutrient-rich jam, jelly, and pickle from Sonneratia apetala fruit
Source: PLoS One. 2024 Dec 4;19(12):e0311846. doi: 10.1371/journal.pone.0311846 (PMC11616811; doi:10.1371/journal.pone.0311846)
Supplement: S2 Table — (DOCX) [file pone.0311846.s002.docx]

Table S2: Comparison of nutrition values of Orange, mango and *S. apetala* fruit’s jam, jelly and pickles.

| Major Elements | Orange Jelly | Orange Jam | Orange Pickle | Mango Jelly | Mango Jam | Mango Pickle | *S. apetala* Jelly | *S. apetala* Jam | *S. apetala* Pickle |
| --- | --- | --- | --- | --- | --- | --- | --- | --- | --- |
|  |  |  |  |  |  |  |  |  |  |
| Na (mg/100gm) | 7.47 |  | 11.0 | 37 | 6.2 | 74.3 | 987.4 | 409.3 | 193.7 |
| K (mg/100gm) | 124.89 | 161.6 | 7.0 | 325.3 | 54.1 | 31.7 | 389.56 | 354.2 | 326.3 |
| Mg (mg/100gm) | 12.54 | 16.1 | 0 | 9 | 64.1 | 4.2 | 1.8 | 1.5 | 0.8 |
| Ca (mg/100gm) | 40.64 | 150 | 8.0 | 10 | 4.8 | 2.2 | 4.54 | 3.49 | 3.1 |
| Fe (mg/100gm) | 0.0 | 1.1 | 0.03 | 0 | 0.3 | 1.8 | 6.7 | 6.7 | 3.2 |
| Mn (mg/100gm) | 0.0 | 0.0 | 0.004 | 0 | 0 | 0 | 2.2 | 1.8 | 1.3 |
| Zn (mg/100gm) | 0.075 | 1.1 | 0.01 | 0.04 | 0.1 | 0.0 | 1.0 | 0.7 | 0.6 |
| Cu (mg/100gm) | 0.025 | 0 | 0.018 | 0.11 | 0 | - | 0.5 | 0.3 | 0.2 |
| P (mg/100gm) | 21.65 | 0.8 | 20.4 | 5.1 | 4.0 | 5.3 | 45.3 | 44.2 | 40.6 |
| Protein (%) | 0.06 | 0.07 | 0.06 | 1.4 | 0.2 | 0.2 | 3.3 | 2.84 | 0.87 |
| Fat (%) | 0.0 | 0.0 | 0.0 | 0.6 | 0.1 | 0.4 | 5.36 | 5.99 | 2.05 |
| Fibre (%) | 4.0 | 4.0 | 0.26 | 2.6 | 0.2 | 0.3 | 0.5 | 19.98 | 4.5 |
| Carbohydrate (%) | 12.0 | 14 | 13.26 | 24.7 | 10.8 | 18.5 | 67.77 | 52.24 | 69.73 |
| Energy (kcal/100 gm) | 245 | 242.6 | 249.5 | 99 | 45.0 | 78.0 | 333 | 287 | 301 |
| Total Suger (gm) | 13 | 13 | 12.0 | 22.5 | 9.7 | 1.0 | 0 | 0 | 0 |
| Vit-C (mg/100gm) | 27.27 | 21.8 | 25.0 | 27.7 | 4.6 | 4.8 | 830.2 | 700.1 | 495.8 |
